# Case Report: Monoclonal Gammopathies of Clinical Significance-Associated Myopathy: A Case-Based Review

**DOI:** 10.3389/fonc.2022.914379

**Published:** 2022-07-14

**Authors:** Hongbin Yu, Du He, Qing Zhang, Bei Cao, Weiping Liu, Yu Wu

**Affiliations:** ^1^ Department of Hematology, West China Hospital, Sichuan University, Chengdu, China; ^2^ Department of Pathology, West China Hospital, Sichuan University, Chengdu, China; ^3^ Department of Cardiology, West China Hospital, Sichuan University, Chengdu, China; ^4^ Department of Neurology, West China Hospital, Sichuan University, Chengdu, China

**Keywords:** MGCS, MGUS, myopathy, amyloidosis, nemaline myopathy

## Abstract

Monoclonal gammopathies of clinical significance (MGCS)-associated myopathy is a group of muscular MGCS-based rare manifestations. It mainly includes amyloid light chain (AL) amyloidosis and sporadic late-onset nemaline myopathy with monoclonal gammopathy of undetermined significance. When myopathy manifests as the initial or sole clinical symptom, it can often be delayed or misdiagnosed as other myopathies. We report the case of a 60-year-old man who initially presented with fatigue and muscle weakness of the symmetric proximal lower limbs. Muscle biopsy did not reveal mononuclear cell infiltration, atrophy, necrosis, or positive Congo red staining results. The results of serum protein electrophoresis and immunofixation electrophoresis were negative. No specific diagnosis was established. After 1 year, the patient was diagnosed with AL amyloidosis after myocardial and fat pad biopsies were performed and myopathy was diagnosed as AL amyloidosis-associated myopathy after reassessment. The patient received CyBorD regime chemotherapy and achieved hematological and organ remission. Therefore, we reviewed the clinical and pathological manifestations of MGCS-associated myopathies. Based on published articles and the present case, we conclude that comprehensive screening for MGCS in unexplained myopathy is essential to avoid misdiagnosis or delayed diagnosis.

## Introduction

Monoclonal gammopathies of clinical significance (MGCS) is a novel term used to describe nonmalignant monoclonal gammopathies that cause prominent diseases. This term is subsequently extended based on the concept of monoclonal gammopathy of renal significance (MGRS) to distinguish between patients diagnosed with monoclonal gammopathy of undetermined significance (MGUS) but actually with clinical symptoms associated with monoclonal gammopathies (1). A group of MGCS disorders presenting with predominant myopathy, including amyloid light chain (AL) amyloidosis-associated myopathy and sporadic late-onset nemaline myopathy with monoclonal gammopathy of undetermined significance (SLONM-MGUS), poses as a diagnostic challenge because of the sensitivity of detection and unawareness of the physicians.

This study describes the case of a patient initially presenting with progressive myopathy who was later diagnosed with AL amyloidosis. The delayed diagnosis of AL amyloidosis-associated myopathy was established after skeletal muscle reassessment. We further reviewed previously published articles focusing on clinical and pathological manifestations of MGCS-associated myopathies.

## Case Presentation

**Figure 1:**
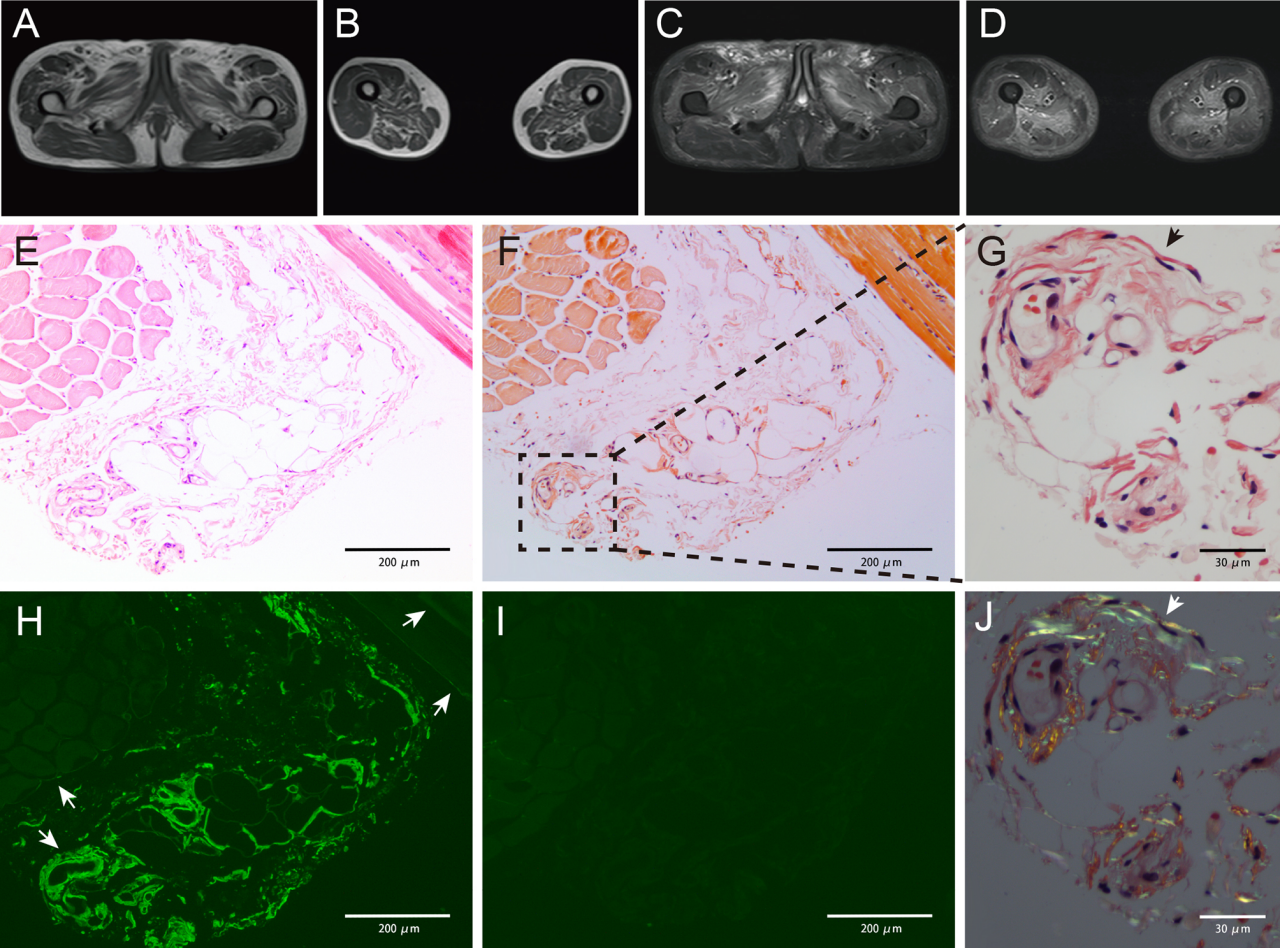
Magnetic resonance imaging results showing abnormality **(A–D)**. Increased signal intensity could be detected in both T1-weighted **(A, B)** and T2-weighted with fat suppression **(C, D)** images, indicating muscle atrophy and edema. The pelvic muscles and inner and posterior muscles of both thighs were affected heavily. Pathology studies of the skeletal muscle showing amyloid deposits **(E–J)**. Muscle biopsy obtained from the patient showing slight myofibers atrophy on hematoxylin and eosin (H&E)-stained section **(E)**. Weak congophilic deposits within the blood vessel walls in Congo red-stained section visualized under light microscopy (**F, G**, black arrow). λ light chain immunofluorescent staining showed a positive staining in blood vessel walls and perimysium (**H**, white arrow), and the perimysium involvement was not detected in Congo red staining. κ light chain immunofluorescent staining showed a negative result **(I)**, indicating a λ light chain restriction. Congo red-stained specimen viewed *via* polarized microscopy revealed positive apple-green birefringence of amyloid deposits (**J**, white arrow). Magnifications (×): 100 in **(E, F, H, I)**; 400 in **(G, J)**.

**Figure 2:**
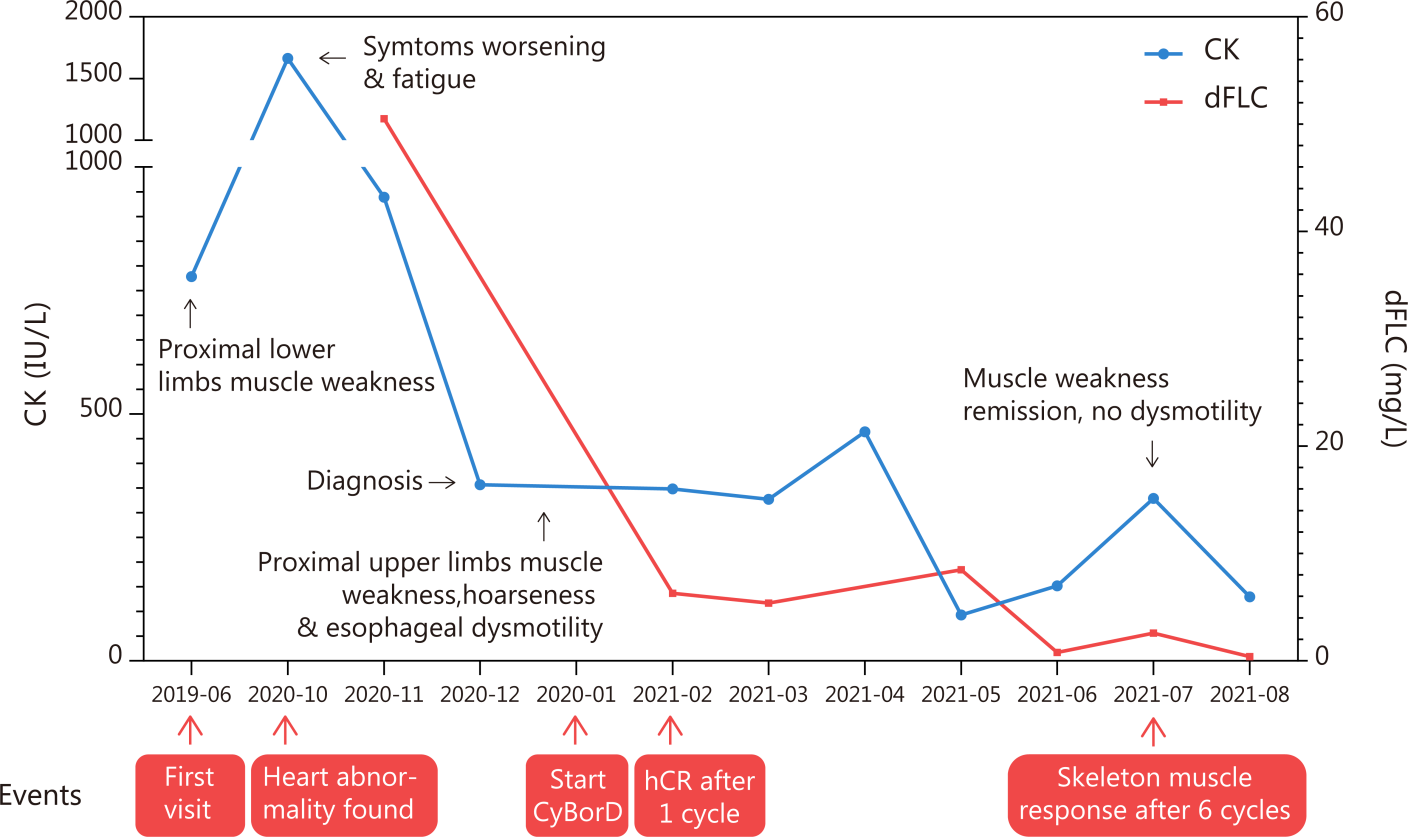
Time and events of the case report. The level of creatine kinase considerably dropped after the initiation of CyBorD regime chemotherapy and is within the normal range at present. Hematological remission was also achieved soon after chemotherapy. Clinical symptoms were alleviated after six cycles of chemotherapy.

A 60-year-old man was hospitalized with muscle weakness of the symmetric proximal lower limbs and fatigue that persisted for 2 months. No myalgia, muscle atrophy, or paresthesia was noted. Physical examinations revealed a decrease in muscle strength during bilateral knee extension, knee flexion, and hip flexion (medical research council [MRC] 4/5). There was no evidence of bulbar, axial, respiratory, or other muscle weakness. The patient did not present with macroglossia, jaw claudication, skin changes, or exertional dyspnea. Infection, previous illness, medication, or family history was not reported. Laboratory examinations indicated that the level of creatine kinase (CK) was 778 U/L and that of lactate dehydrogenase (LDH) was 269 U/L. Autoantibodies (antinuclear antibodies; anti-neutrophil cytoplasmic antibodies; rheumatoid factors; myositis-specific antibodies, including anti-aminoacyl-tRNA synthetase antibodies; and myositis-associated antibodies, including antibodies against PM-Scl, U1RNP, Ku, Ro, and cN-1A) were not detected, and the results of serum protein electrophoresis (SPE) and immunofixation electrophoresis (IFE) were negative for the monoclonal protein (M protein). Magnetic resonance imaging (MRI) revealed atrophy and edema in proximal lower limbs and pelvic muscles (**Figures 1A–D**). Electromyography (EMG) showed that the motor and sensory nerve conduction results and F-waves were normal in both the upper and lower extremities. The biceps brachii and vastus medialis showed severe fibrillation waves and positive sharp waves as well as short-duration and low-amplitude motor unit action potentials and early recruitment, indicating myopathy with membrane irritability in the proximal muscles of both the upper and lower extremities. The right quadriceps biopsy revealed a few slightly atrophic myofibers but no mononuclear cell infiltration. The results of Congo red staining were negative. The patient was diagnosed with “undetermined myopathy” and received supportive therapy without improvement. His lower limb weakness and fatigue progressively deteriorated. Muscle weakness progressed to his proximal upper limbs, and his voice became hoarse in the following year. The patient was then referred to our hospital. He walked into the hospital with a slow quadriceps gait and was unable to stand up from a chair without assistance. Physical examinations revealed a decrease in muscle strength during bilateral elbow flexion (MRC 4/5). The following laboratory examination results were obtained: CK, 1665 (19–226) IU/L; CK-MB, 34.37 (<4.94) ng/ml; LDH, 397 (120–250) IU/L; N-terminal-pro hormone brain natriuretic peptide (NT-proBNP), 4,534 (0–227) ng/L; cardiac troponin T, 93.3 (0–14) ng/L; and aspartate aminotransferase, 57 (<40) IU/L. Echocardiography revealed that his interventricular septum (IVS) was 17 mm and ejection fraction (EF) was 40%. M spike was undetectable on SPE. IFE exhibited low levels of immunoglobulin M (IgM) without a monoclonal band. The serum-free light chain (FLC) levels were 14.3 (6.7–22.4) mg/L for κ and 64.8 mg/L (8.3–27 mg/L) for λ. The plasma cell count was 4.5% in the bone marrow (BM) smear, and flow cytometric analysis revealed a clonal plasma cell count of 3.3% in the BM. The patient did not meet the diagnostic criteria for multiple myeloma. Myocardial and abdominal subcutaneous fat pad aspiration biopsies were performed. Congo red staining revealed congophilic deposits in myocardium and the fat pad, and electron microscopic examinations exhibited amyloid fibril deposits in the samples obtained from both sites. Tc-99m pyrophosphate scintigraphy results were negative. Further immunohistochemical examination of the myocardial sections confirmed the λ restriction and negativity of transthyretin, ruling out amyloidogenic transthyretin (ATTR) amyloidosis. The patient was thus diagnosed with AL amyloidosis with cardiac involvement (Mayo 2012 Stage III). He received cyclophosphamide, bortezomib, and dexamethasone (CyBorD) chemotherapy and achieved complete hematologic remission after the first cycle. The skeletal muscle sections from the previous paraffin specimens were reanalyzed. Congo red staining revealed a weak congophilic deposition in the blood vessel walls in the connective tissue, and the apple-green birefringence of amyloid deposits was further confirmed using a polarized microscope at the same site. Immunofluorescence staining showed a λ light chain restriction in both blood vessel walls and perimysium, which is strongly positive in blood vessel walls but weakly positive in the perimysium (**Figures 1E–J**). The patient was then diagnosed with AL amyloidosis-associated myopathy. He reported improvement in muscle strength, esophageal dysmotility, and hoarseness after receiving six cycles of chemotherapy. A considerable reduction in CK level was detected during treatment (**Figure 2**). After the seventh cycle, a very good partial remission was achieved in cardiac response (NT-proBNP, 1735 ng/L). Owing to the cardiac dysfunction, the patient did not meet the criteria for autologous hematopoietic stem cell transplantation (ASCT) and is currently under follow-up. Following chemotherapy, no clonal plasma cells were detected in the BM. The patient’s IVS was 16 mm, and his EF was 47% on echocardiography. The patient reported that he has returned to his normal routine.

## Discussion and Literature Review

MGCS is a novel concept that has emerged in recent years. It describes monoclonal gammopathy with targeted organ damage based on corresponding symptoms and comprises a spectrum of disorders. Although, to the best of our knowledge, there is no consensus regarding the diagnosis of the concise diseases cataloged in MGCS, the American Society of Hematology Education Program has published a list of diseases belonging to the MGCS group (1). Among those diseases, MGCS-associated myopathy, including AL amyloidosis-associated myopathy and SLONM, is well established, although often overlooked.

### AL Amyloidosis-Associated Myopathy

AL amyloidosis is caused by the misfolding and deposition of immunoglobulin light chains produced by a dangerous small B-cell or plasma cell clone as amyloid fibrils, causing systematic organ dysfunction (2). Skeletal muscles can be involved, and the incidence of myopathy caused by AL amyloidosis is very low (approximately 1.5% [51/3,434] in patients with AL amyloidosis) (3). Patients with skeletal muscle involvement generally present with muscle weakness (frequently only in proximal muscles but sometimes in both proximal and distal muscles). Myalgia, muscle atrophy, dysphagia, hoarseness, macroglossia, pseudohypertrophy, and jaw claudication usually are “red flags” for AL amyloidosis-associated myopathy; pseudohypertrophy is a distinctive feature of AL amyloidosis as compared with other MGCS-associated myopathies (3, 4). The disease can be easily misdiagnosed as idiopathic inflammatory myositis (IIM) or diagnosed nonspecifically because of its shared clinical features with IIM (3). In the present case, the patient had a 53% probability of polymyositis based on a widely consensual probability-score model for IIM before AL-associated myopathy was confirmed (which is defined as “possible IIM,” according to the model) (5). In laboratory examinations, in addition to monoclonal immunoglobulin revealed on SPE, IFE, or FLC assay, CK level can increase above the upper limit but generally not too high (nearly one-third of the patients had CK level above the upper limit of the normal level) (3). The diagnosis of AL amyloidosis depends on the findings of amyloidosis caused by the deposition of monoclonal immunoglobulin light chains in organs. The results of hematoxylin and eosin (H&E) staining of the skeletal muscle section may appear normal. Atrophic, regenerating, and necrotic myofibers may also be observed (3). The results of Congo red staining and electron microscopic examinations can reveal amyloid deposition in the intramuscular blood vessel walls and connective tissues (perimysium and endomysium), occasionally encasing muscle fibers (3, 6), which is a characteristic feature distinguishing AL amyloidosis-associated myopathy from the other myopathies. The type of amyloid should be verified using immunostaining or mass spectrometry because amyloidosis varies across muscles (ATTR or isolated amyloid myopathies) (6). The involved muscle may exhibit edema and atrophy on MRI (7, 8), similar to that noted in the case of IIM. EMG often shows nonspecific myopathic changes, which include spontaneous activity (fibrillation potentials and positive sharp waves) and typical myopathic motor unit action potentials (early recruitment of short-duration and low-amplitude motor unit action potentials); these changes are different from the features of patients with peripheral neuropathy, which is important in differential diagnosis, although neuropathy is often observed in AL amyloidosis-associated myopathy due to direct nerve involvement (8–12). The key to treating the AL amyloidosis-associated myopathy is achieving complete hematologic remission by eliminating the M proteins. The recommended chemotherapy regimens include daratumumab-CyBorD and CyBorD. Additionally, ASCT should be considered if the patient is eligible. The overall survival of AL amyloidosis depends on the remission depth of the hematological response and damage of the involved organs, particularly the heart. Mayo Clinic has reported that the predicted overall survival period of patients with AL amyloidosis-associated myopathy without cardiac involvement is 102 months (3).

### SLOMN-MGUS

Sporadic late-onset nemaline myopathy (SLONM) is a term that describes a subtype of nemaline myopathy, which is distinguished from the nemaline myopathy characterized by genetic mutations and usually develops at a young age (13). Some patients with SLONM were found to be associated with monoclonal gammopathy or HIV infection. SLONM with monoclonal gammopathy was termed “SLONM-MGUS,” which has recently been regarded as an MGCS-associated myopathy (1). SLOMN-MGUS is characterized by rod-shaped structures (nemaline rods) in myofibers observed *via* histochemical or electron microscopic examinations; the findings are consistent with those of other types of nemaline myopathy. Modified Gomori trichrome staining is a significant method that reveals the nemaline rods. To date, the mechanism underlying the disease remains little understood. The rods are considered to arise from myofibers’ Z-bands on the basis of histopathological finding, which reportedly results from insufficient autophagy (14–17). It was reported that nemaline rods only reflected secondary pathological changes due to various primary damages. In patients with SLOMN-MGUS, the M protein was found to be not involved in the composition of nemaline rods on the basis of the negative immunostaining result of heavy or light chain, which indicated that nemaline rods in SLOMN-MGUS may result from M protein damage (14, 18–20). Furthermore, the number of rods may be related to the progression or remission of clinical symptoms (21, 22). The clinical features of patients with SLONM-MGUS are generally muscle weakness of the axial and proximal upper or lower limbs, with occasional distal muscle weakness and neck extensor weakness (dropped head). Nearly all patients exhibited respiratory muscle weakness. Dyspnea, which distinguishes SLOMN-MGUS from AL amyloidosis-associated myopathy and results in death, is common in patients with SLOMN-MGUS (13). Muscle atrophy, myalgia, and dysphagia may also occur. Dilated cardiomyopathy has also been reported; however, its involvement remains uncertain (16, 23). The results of SPE, IFE, and FLC assay can confirm the diagnosis of monoclonal gammopathy. The CK level is primarily normal or slightly increased, which is similar to a feature of AL amyloidosis-associated myopathy (13). Regarding pathological findings, H&E staining may reveal the normality or slight atrophy of myofibers. Inflammatory infiltration, myofibrillar disintegration, core-like area, and lobulated fiber may also be detected at times (13, 17). MRI may reveal atrophy, fatty degeneration, and edema (16, 24, 25). Myopathic changes, including spontaneous activity (fibrillation potentials and fasciculation potentials) and myopathic motor unit action potentials (early recruitment of short-duration motor unit potentials mixed with long-duration motor unit potentials), can be detected on EMG. However, mixed changes and pure neurogenic changes may be observed (13, 17, 23). Patients with SLONM-MGUS are considered to exhibit rapid disease progression and worse prognosis than those with SLOMN without MGUS. However, a recent retrospective study revealed that the overall survival of patients may not be affected by monoclonal gammopathy (13, 23, 26). Nonchemotherapeutic strategy (immunosuppressive agents, intravenous immunoglobulins, and blood purification), chemotherapeutic strategy aimed at eliminating the M protein, and ASCT were used to achieve myopathy remission for patients with SLONM-MGUS (27, 28). Both organ response (13 [52%] vs. 24 [86%]) and overall survival were superior in patients who received chemotherapy and ASCT than those who received nonchemotherapeutic treatment (29). Thus, SLONM-MGUS is considered an MGCS. However, given the small sample size and lack of a prospective study, the first-line treatment remains highly debatable (30). Some physicians suggest that the term SLOMN-MP (M protein) be used instead of SLOMN-MGUS as patients with this disease have clinical significance in the muscles.

### Can Other MGCS/Multiple Myeloma Result in Myopathy?

In addition to AL amyloidosis and SLOMN-MGUS, a few cases of other M protein-associated myopathies have been reported. In rare cases, POEMS syndrome has been associated with myopathy, and the muscular manifestation resulted from an inflammatory mechanism rather than innervation dysfunction. Furthermore, chemotherapy or immunosuppressive therapy could reverse the myopathy (31, 32). However, given the limitations of pathological methods and scarcity of cases, the correlation between POEMS syndrome and myopathy remains debatable. Multiple myeloma may combine with AL amyloidosis-associated myopathy or SLONM-MGUS (33). Meanwhile, given that multiple myeloma accounts for the majority of plasma cell neoplasms, myeloma with muscular infiltrations, particularly those that have relapsed as intramuscular extramedullary plasmacytomas, should not be overlooked (34, 35). In summary, although a few case reports suggest that other MGCS or multiple myeloma can cause myopathy, the evidence is insufficient to establish a clear correlation between the other MGCS/multiple myeloma and myopathy based on current knowledge (36, 37). Nonetheless, physicians should be aware of the potential associations. **Table 1** summarizes the characteristics of AL amyloidosis-associated myopathy and SLOMN-MGUS manifestations.

**Table 1:** Features of MGCS-associated myopathies.

	Frequent Clinical Features of Myopathy	Laboratory Findings	Imaging Features	EMG	Pathogenic Findings
AL amyloidosis	Muscle weakness (frequently proximal muscles), myalgia, muscle atrophy, dysphagia, hoarseness, macroglossia, and jaw claudication	M protein; creatine kinase level is mostly normal, may elevate but generally not too high	Atrophy, edema, and fatty degeneration	Spontaneous activity and myopathic motor unit action potentials	Generally normal in H&E staining; atrophy, regeneration, and necrosis in myofibers sometimes; Congo red staining results are positive; light chain immunostaining results are positive; electron microscopic examinations can detect amyloid
SLONM-MGUS	Muscle weakness (frequently axial and proximal muscles), dropped head, muscle atrophy, myalgia, dysphagia, and dyspnea			Myopathic changes include spontaneous activity and myopathic motor unit action potentials; neuropathic changes may also be present	Generally normal in H&E staining; atrophy, regeneration, and necrosis in myofibers sometimes; nemaline rods may be detected on modified Gomori trichrome staining and electron microscopic examinations

**Figure 3:**
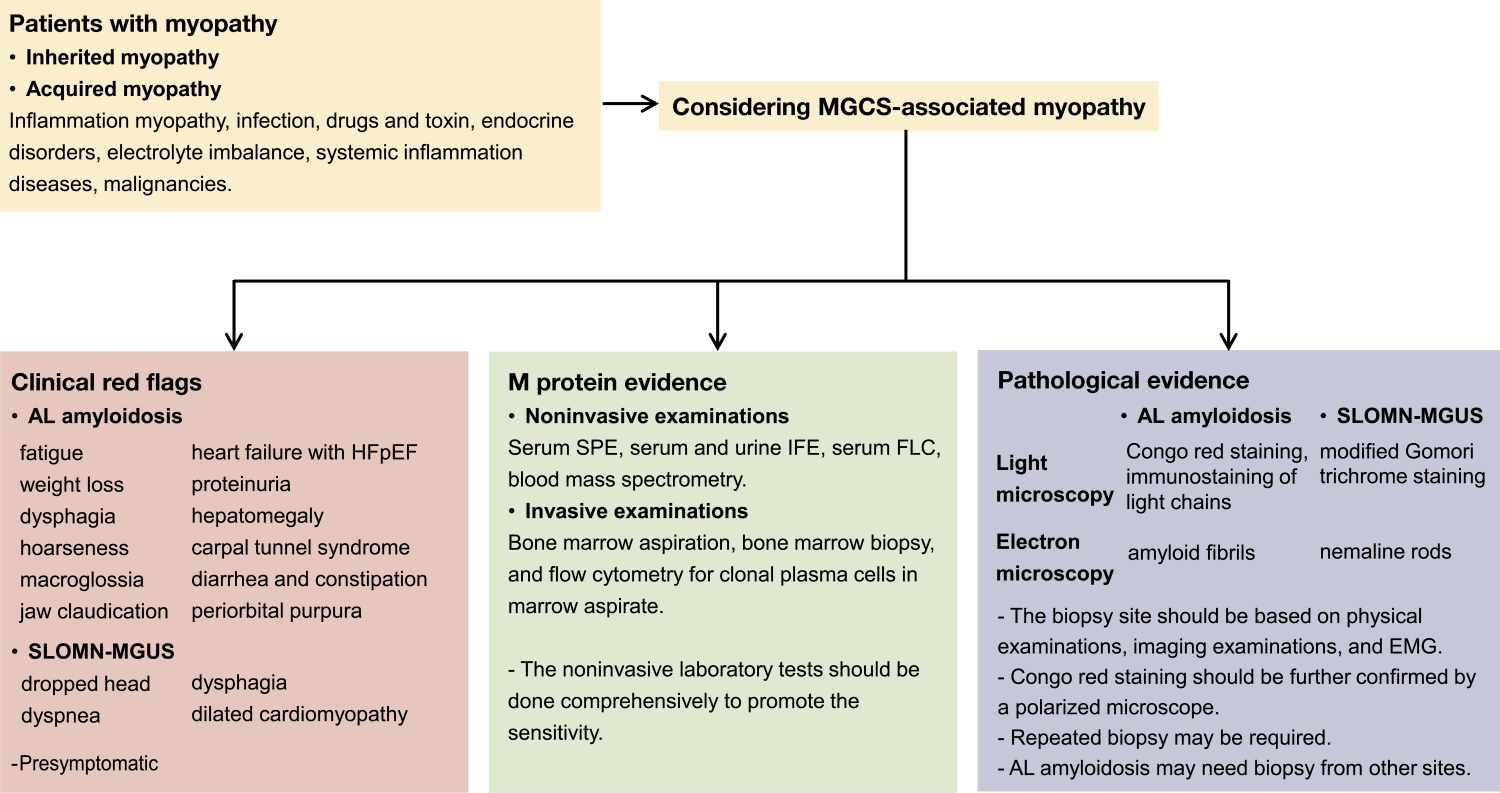
Workup for patients suspected of MGCS-associated myopathy. Clinical red flags if presented may suggest AL amyloidosis or SLOMN-MGUS. Systemic damages are common in AL amyloidosis, and some presymptomatic signs should also be noted in AL amyloidosis. M protein screening (comprehensive noninvasive and invasive bone marrow examinations) is required for diagnosis. Pathological evidence is crucial for MGCS-associated myopathy diagnosis. Special staining methods such as Congo red staining for AL amyloidosis and modified Gomori trichrome staining for SLOMN-MGUS are useful. Light chain immunostaining is only useful for AL amyloidosis. Electron microscopy is indispensable for differential diagnosis.

The differential diagnosis of MGCS-associated myopathy is essential in clinical practice, and pathological findings are key to an accurate diagnosis. Delayed or misdiagnosis is common in MGCS-associated myopathy (Mayo Clinic reported that 40% AL amyloidosis-associated myopathy patients who underwent muscle biopsies were misdiagnosed before arrival at their center) (3). The median duration from the onset of initial symptoms to the diagnosis of disease in patients with AL amyloidosis-associated myopathy is 23 months and that for patients with SLONM is 35 months (3, 23). Comprehensive M protein examinations, pathological diagnosis with considerable expertise, and screening for systemic presentations could prevent misdiagnosis. In this case, despite the nondetection of the monoclonal protein on SPE and IFE, an FLC assay should be performed. Only Congo red staining was performed using muscle specimens initially, and the staining results were too faint to be observed (**Figures 1G, J**), which might have resulted in a delayed diagnosis outside our center. Furthermore, the patient’s systemic evaluation is insufficient. This case demonstrated that the negative results obtained after SPE and IFE performed using serum and urine samples do not rule out the possibility of AL amyloidosis or other MGCS. Negative results may be obtained after performing serum IFE and urine IFE in 20%–24% and 12% of patients diagnosed with AL amyloidosis, respectively. FLC assay has a higher sensitivity (88%–98%) for the screening of M protein (38–40). The sensitivity is remarkable (99.8%) if these noninvasive examinations (serum IFE and FLC and urine IFE) are performed comprehensively. These examinations are convenient and crucial for the diagnosis of MGCS (36). Recently, a novel blood mass spectrometry method has been reported for detecting the M protein with a higher sensitivity than FLC (41, 42). To add more clinical information, invasive BM examinations such as BM aspiration, BM biopsy, and flow cytometry of clonal plasma cells present in marrow aspirate must be performed in patients with a high suspicion of MGCS. Pathological evidence is the gold standard for making a diagnosis. Thus, the tissue involved must be assessed carefully. The combination of special staining, immunostaining, and electron microscopic examinations (which can directly reveal amyloid fibrils and nemaline rods) can improve the detection sensitivity. Mass spectrometry should be considered in selected patients. Moreover, the choice of muscle biopsy site should be based on previous examinations to improve the sensitivity of the pathological examination. Alternative organ or fat pad biopsy, repeated muscle biopsy, or reassessment is sometimes required to make a final diagnosis (23). Furthermore, for suspected MGCS-associated myopathy, screening for systemic manifestations is crucial. The screening should include the presymptomatic signs of organ involvement (unexplained elevated level of NT-proBNP, high level of alkaline phosphatase, and presence of albuminuria). Meanwhile, reassessing the initial diagnosis and dynamically evaluating the disease progression throughout the disease course are essential. A multidisciplinary team may help with comprehensive assessment. Herein, we have summarized the workup for patients with myopathy who are being considered for MGCS (**Figure 3**).

## Conclusions

MGCS-associated myopathy is a rare condition. Awareness regarding its clinical manifestations, comprehensive M protein detection methods, pathology techniques, and diagnostic expertise are important for early and precise diagnosis.

## Data Availability Statement

The original contributions presented in the study are included in the article/supplementary material. Further inquiries can be directed to the corresponding author.

## Ethics Statement

The studies involving human participants were reviewed and approved by the Institutional Review Board of West China Hospital, Sichuan University. The patients/participants provided their written informed consent to participate in this study.

## Author Contributions

HY collected clinical information, wrote the manuscript, and conducted the review. DH and WL conducted the pathological review of the patient, including the reassessment of the skeleton muscle specimen. QZ conducted the initial clinical evaluation and confirmed cardiac involvement. BC confirmed and compared the EMG results. YW contributed to the conception and design of the study, manuscript writing, and final review of the manuscript. All authors contributed to the article and approved the submitted version.

## Conflict of Interest

The authors declare that the research was conducted in the absence of any commercial or financial relationships that could be construed as a potential conflict of interest.

## Publisher’s Note

All claims expressed in this article are solely those of the authors and do not necessarily represent those of their affiliated organizations, or those of the publisher, the editors and the reviewers. Any product that may be evaluated in this article, or claim that may be made by its manufacturer, is not guaranteed or endorsed by the publisher.
